# Responses of photosystem to long-term light stress in a typically shade-tolerant species *Panax notoginseng*

**DOI:** 10.3389/fpls.2022.1095726

**Published:** 2023-01-12

**Authors:** Zhu Cun, Xiang-Zeng Xu, Jin-Yan Zhang, Sheng-Pu Shuang, Hong-Min Wu, Tong-Xin An, Jun-Wen Chen

**Affiliations:** ^1^ College of Agronomy & Biotechnology, Yunnan Agricultural University, Kunming, China; ^2^ Key Laboratory of Medicinal Plant Biology of Yunnan Province, Yunnan Agricultural University, Kunming, China; ^3^ National & Local Joint Engineering Research Center on Germplasm Innovation & Utilization of Chinese Medicinal Materials in Southwestern China, Yunnan Agricultural University, Kunming, China; ^4^ Research Center for Collection and Utilization of Tropical Crop Resources, Yunnan Institute of Tropical Crops, Xishuangbanna, China

**Keywords:** photosynthesis, chlorophyll fluorescence, photosystem, photoprotection, *Panax notoginseng*

## Abstract

Photosynthetic adaptive strategies vary with the growth irradiance. The potential photosynthetic adaptive strategies of shade-tolerant species *Panax notoginseng* (Burkill) F. H. Chen to long-term high light and low light remains unclear. Photosynthetic performance, photosynthesis-related pigments, leaves anatomical characteristics and antioxidant enzyme activities were comparatively determined in *P. notoginseng* grown under different light regimes. The thickness of the upper epidermis, palisade tissue, and lower epidermis were declined with increasing growth irradiance. Low-light-grown leaves were declined in transpiration rate (Tr) and stomatal conductance (Cond), but intercellular CO_2_ concentration (*C*_i_) and net photosynthesis rate (*P*_n_) had opposite trends. The maximum photo-oxidation ${\text{P}}_{700}^{+}$ (*P*_m_) was greatly reduced in 29.8% full sunlight (FL) plants; The maximum quantum yield of photosystem II (*F*_v_/*F*_m_) in 0.2% FL plants was significantly lowest. Electron transport, thermal dissipation, and the effective quantum yield of PSI [Y(I)] and PSII [Y(II)] were declined in low-light-grown plants compared with high-light-grown *P. notoginseng*. The minimum value of non-regulated energy dissipation of PSII [Y(NO)] was recorded in 0.2% FL *P. notoginseng*. OJIP kinetic curve showed that relative variable fluorescence at J-phase (*V*_J_) and the ratio of variable fluorescent *F*_K_ occupying the *F*_J_-*F*_O_ amplitude (*W*_k_) were significantly increased in 0.2% FL plants. However, the increase in *W*_k_ was lower than the increase in *V*_J_. In conclusion, PSI photoinhibition is the underlying sensitivity of the typically shade-tolerant species *P. notoginseng* to high light, and the photodamage to PSII acceptor side might cause the typically shade-tolerant plants to be unsuitable for long-term low light stress.

## Introduction

Light plays an indispensable role in the growth and development of plants (de Wit et al., 2016). However, light fluctuates over short (seconds) and long (hours, days, seasons) timescales in natural condition, making it highly heterogeneous (Townsend et al., 2018a; Townsend et al., 2018b; Townsend et al., 2018c). Two species have emerged under long-term evolutionary processes, markedly different in their light demands: the light-demanding species and shade-tolerant species (Mathur et al., 2018). The light-demanding species such as *Spinacea oleracea* and *Oryza sativa*, show high values of maximum CO_2_ assimilation rate (*P*_max_), non-photochemical quenching (NPQ) and electron transport rates (Osmond et al., 2021; Wei et al., 2021). The shade-tolerant species such as *Picea glauca*, *Abies balsamea* and *Abies lasiocarpa* exhibit low *P*_max_, light saturating/compensation points (LSP/LCP) and dark respiration rates (*R*_d_) (Valladares and Niinemets, 2008). Several studies have shown that the shade-tolerant species not only need to improve the efficiency of light energy utilization under low light, but also to strengthen the dissipation of excess light energy under high light condition (Kim et al., 2020; Ware et al., 2020). The quantum yield of photosystem II (PSII), photosynthetic electron transport and photochemical quenching are increased in shade-tolerant species *Bletilla striata* exposed to a sudden transition from low to high light (Yang et al., 2019a). PSII activity is reduced in the shade-tolerant species *Anacardium excelsum* and *Virola surinamensis* grown under high light (Barth et al., 2001). Meanwhile, low photosystem I (PSI) activity has been recorded in the shade-tolerant species *Psychotria henryi* and *Psychotria rubra* exposed to high light (Huang et al., 2015; Huang et al., 2017). Therefore, more research is needed in the PSI of shade-tolerant plant to elucidate its potential mechanism of PSI in response to light stress.

Long-term light stress induces photoinhibition and even photodamage of plants when absorbed light energy would temporarily exceed the need for photosynthesis (Niyogi and Truong, 2013; Kono and Terashima, 2014). Light stress protection mechanisms include chloroplastic reactive oxygen species (ROS) scavenging, chloroplast and stomatal movement (Shi et al., 2022). For example, high-light-grown *Triticum aestivum* leaves reduced ROS-mediated side-effects by increasing the activity of catalase (CAT) and superoxide dismutase (SOD, Szyma´nska et al., 2017). Low light could induce rapid stomatal opening to enhance photosynthesis and photorespiration of *Phaseolus vulgaris* (Pastenes et al., 2005). Meanwhile, photosynthetic apparatuses (PSI and PSII) have evolved a variety of photoprotective strategies to dissipate excess light energy (Bosch et al., 2015). NPQ is considered to be the most efficient strategy for thermal dissipation of excess light energy (Han et al., 2022). The increase in NPQ with the enhancement of light intensity has been recorded in the shade-tolerant species *Coffea arabica* and *Tradescantia sillamontana* (Martins et al., 2014; Mishanin et al., 2016; Mishanin et al., 2017). Nevertheless, plants might improve the utilization of excess light energy by enhancing electron transport (Kalmatskaya et al., 2020), as has been recorded in the shade-tolerant species *Vanda* sp. (Sma-Air and Ritchie, 2020). Meanwhile, cycle electron flow (CEF) is an efficient pathway for utilizing excess light energy (Tikhonov, 2013). The CEF-dependent generation of the proton gradient (ΔpH) across the thylakoid membrane not only stimulates ATP synthesis but also protects PSII from photoinhibition through activating NPQ and stabilizing oxygen-evolving complexes (Theune et al., 2021). Moreover, activation of CEF-PSI can also prevent PSI from photoinhibition and photooxidative damage through alleviating the over-reduction of PSI acceptor side and reducing the synthesis of superoxide anions in PSI (Sagun et al., 2019; Yang et al., 2019a; Yang et al., 2019b). Photooxidative damage is avoided in the shade-tolerant species such as *Vanilla orchid*, *Neobalanocarpus heimii* and *Lepisanthes senegalensis* through enhancing CEF around PSI when it is exposed to high light (Kang et al., 2020; Ko et al., 2020). Nevertheless, it is still unknown about a relationship between the photoprotective strategies and the sensitivity of the shade-tolerant species to high light.

*Panax notoginseng* (Burkill) F. H. Chen (Sanqi in Chinese) is a perennial Chinese herb (the *Panax* genus, Araliaceae), which is a typically shade-tolerant species (Zhang et al., 2020). Full light (FL) of 9.6%-11.5% was found to be the most suitable growth light environment for *P. notoginseng* (Zuo et al., 2014; Kuang et al., 2014a; Kuang et al., 2014b; Kuang et al., 2015). Net photosynthesis rate (*P*_n_), stomatal conductance (Cond), and transpiration rate (Tr) are significantly inhibited in excessive-shading-grown *P. notoginsen* (Xu et al., 2018). Meanwhile, the thermal dissipation and carboxylation efficiency are improved in high-light-grown *P. notoginseng*; correspondingly, the efficiency of PSII photochemistry is decreased in low-light-grown counterpart (Chen et al., 2014; Chen et al., 2016). In addition, Huang et al. (2018a) have found that PSI photoinhibition did not occur in high-light-grown *P. notoginseng*, but LEF (linear electron flow) declined due to a decrease in PSII activity. The results are contrary to the findings that high light might induce the irreversible damage to PSII and the moderate photoinhibition to PSI in *P. notoginseng* (Wu et al., 2021). However, it is still unclear whether high-light induce irreversible damage to photosystem in shade-tolerant species. Thus, photosynthetic adaptive strategies in shade-tolerant species grown under light stress need to be further understood. In the present study, photosynthetic performance, photosynthesis-related pigments, leaves anatomical characteristics and antioxidant enzyme activities were comparatively determined in the shade-tolerant species *P. notoginseng* grown under a light gradient. It has been hypothesized that: (1) PSI photoinhibition might underlie the sensitivity of *P. notoginseng* to high light; (2) Enhanced photosynthetic electron transport and moderate PSII photoinhibition might be the photoprotective strategies under high light; (3) The acceptor side of PSII were damaged in *P. notoginseng* were long-term exposed to low light; (4) The photodamage of PSI could be avoided by activating cycle electron transport around PSI in *P. notoginseng* grown under long-term light stress.

## Materials and methods

### Plant materials and growth condition

The pot experiment was carried out from Januray in Wenshan Miao Xiang *P. notoginseng* Technology Park (23°05′N, 104°03′E), Yunnan, China. The healthy two-year-old rhizome of *P. notoginseng* were cultivated in plastic pots (30 cm × 25 cm × 25 cm), with each containing 3 rootstocks. Total photon exposure per day in screened growth house for seven treatments was equivalent to 29.8%, 11.5%, 9.6%, 5.0%, 3.6%, 1.4% and 0.2% of that in the full sunlight (FL), respectively. **Figure S1** shows the diurnal variation of photosynthetic photon flux density (PPFD) under seven light treatments, respectively. 210 pots were used for each light intensity regimes, and a total of 1470 pots were arranged (*n* = 7). Polyoxin and agricultural streptomycin were used to control pests and diseases. In September, the youngest fully expanded functional leaf on each treatment at the maximum nutritional period from pot planting was used for the determination of photosynthetic performance, photosynthesis-related pigments, leaves anatomical characteristics and antioxidant defense system analysis.

### Chlorophyll content measurements

Chlorophyll (Chl) was extracted as described by Pérez-Patricio et al. (2018). A LI-3000 leaf-area meter (Li-Cor, USA) was used to determine leaf area. 0.5 g of fresh leaves were immersed in a 15 mL extraction mixture [99% acetone was mixed with ethanol (2:1 v/v)]. 3 h of standing in the dark were followed by a 10 min centrifugation at 3000 *g*. Absorbance readings were performed at wavelengths of 665 nm and 649 nm. Chl *a* and *b* content were calculated based on the method of Gu et al. (2016). Total Chl content was the sum of Chl *a* and *b*.

### Measurement of gas exchange

Gas exchange measurements were performed between 09:00 and 11:00 on fully expanded function leaves using an LI-6400XT portable photosynthesis system equipped with a 6400-40 leaf chamber (LI-Cor, UAS). Leaf temperature was maintained at 25°C in the chamber. PPFD was 500 μmol·m^-2^·s^-1^ and CO_2_ concentration was adjusted to 400 mmol·mol^-1^ with a mixture. After equilibration to a steady state, net photosynthesis rate (*P*_n_), stomatal conductance (Cond), transpiration rate (Tr), and intercellular CO_2_ concentration (*C*_i_) were recorded.

### Chlorophyll fluorescence and P700 measurements

Dual-PAM 100 chlorophyll (Chl) fluorometer (Walz, Germany) was used to determine PSI and PSII Chl fluorescence parameters at 25°C. Seven plants were dark-adapted for 20 min, and both PSI and PSII parameter were monitored to record Chl fluorescence and P700 state. Then leaves were light-adapted at 172 μmol·m^-2^·s^-1^ for 20 min. Subsequently, PSI and PSII parameters were determined after 120 s exposure to each light intensity (0, 36, 94, 132, 172, 272, 421, and 611 μmol·m^-2^·s^-1^; PPFD, photosynthetic photon flux density). The chlorophyll fluorescence parameters were calculated as follows (Genty et al., 1989; Oxborough and Baker, 1997; Hendrickson et al., 2004): *F*_v_/*F*_m_ = (*F*_m_ - *F*_o_)/*F*_m_; Y(II) = (*F*_m_`-*F*_s_)/*F*_m_`; Y(NO) = *F*_s_/*F*_m_; NPQ = (*F*_m_ - *F*_m_`)/*F*_m_`; 1 – *qP* = (*F*_s_ - *F*_o_`)/(*F*_m_` - *F*_o_`); Y(NPQ) = *F*_s_/*F*_m_` - *F*_s_/*F*_m_. *F*_o_ and *F*_o_` were the minimum fluorescence after dark- and light- adaptation, respectively; *F*_m_ and *F*_m_` were the maximum fluorescence after dark- and light-adaptation, respectively; and *F*_s_ was the dark-adapted steady-state fluorescence. *F*_v_/*F*_m_ was the maximum quantum yield of photosystem II. Y(II) was the effective quantum yield of PSII photochemistry. Y(NO) and Y(NPQ) were the yield of non-regulated and regulated energy dissipation of PSII, respectively. NPQ was the non-photochemical quenching in PSII. 1-*qP* was the redox poise of the primary electron acceptor of PSII.

P700 redox state was calculated by the saturation pulse (600 ms, 10000 μmol·m^-2^·s^-1^) method (Klughammer and Schreiber, 2008). The ${\text{P}}_{700}^{+}$ signals (*P*) may vary between a minimal (P700 fully reduced) and a maximal level (P700 fully oxidized); the maximum photo-oxidation ${\text{P}}_{700}^{+}$ (*P*_m_) and *P*_m_` were ascertained the application of a saturation pulse after pre-illumination with far-red light and actinic light, respectively (Huang et al., 2010; Yamori et al., 2016; Takagi et al., 2017). The chlorophyll fluorescence parameters were determined by Klughammer and Schreiber (2008) method: Y(I) = (*P*_m_` - P)/*P*_m_; Y(ND) = *P/P*_m_; Y(NA) = (*P*_m_ - *P*_m_`)/*P*_m_. Y(I) was the effective quantum yield of PSII; Y(ND) and Y(NA) were the donor side and acceptor side limitation of PSI, respectively.

Photosynthetic electron flows through PSI and PSII were analyzed according to the method described by Huang et al. (2012a); Huang et al. (2017); Huang et al. (2019): ETRII = Y(II) × PPFD × 0.84 × 0.5; ETRI = Y(I) × PPFD × 0.84 × 0.5. ETRI was the electron transport rate of PSI; ETRII was the electron transport rate of PSII. Furthermore, the electron transport rate of cyclic electron flow around PSI was estimated as ETRI - ETRII; the quantum yield of cyclic electron flow around PSI was estimated as Y(I) – Y(II), or expressed as Y(I)/Y(II) (Miyake et al., 2005; Fan et al., 2016; Sagun et al., 2019).

### Measurement of OJIP kinetic curve

Fast Chl fluorescence measurements were conducted by a pulse-amplitude modulation (PAM) fluorometer (PAM-2500, Walz, Germany). After a dark adaptation for 4 h, Chl fluorescence transient curves (OJIP transients) were inducted by a red light (652 nm) of 3000 μmol·m^-2^·s^-1^ by the PAM-2500 through an array of light-emitting diodes. Cha *a* fluorescence emission inducted by the strong light pulses was measured and digitized between 10 μs and 320 ms (Kanutsky curve; Kautsky and Hirsch, 1931). Meanwhile, four characteristic levels of fluorescence yield can be distinguished in a plot with logarithmic time scale: *F*_o_, *I*_1_, *I*_2_ and *F*_m_ (alternatively also denoted O, J, I and P; Schreiber et al., 1986; Schreiber et al., 1989),. The *F*_o_ - *I*_1_ (or O-J) phase of the transient directly reflects the closure of PSII reaction centers by charge separation (Q_A_-reduction). The initial rate of increase of this phase is proportional to the applied light intensity (photochemical phase). At a given light intensity, the initial rate provides a relative measure of the optical absorption cross-section of PSII. The *I*_1_- *I*_2_ - *F*_m_ (or J-I-P) phases of the transient reflect the reduction of the rest of the electron transport chain defined mainly by the reduction of the plastoquinone pool and the acceptor side of PSI; the rate of which is limited by dark reactions (thermal phase) (Schreiber and Klughammer, 2021). The point of time corresponding to 300 μs on the OJIP kinetic curves was defined as the “K” characteristic points (Eggenberg et al., 1995; Strasser et al., 2000; Strasser et al., 2004). The OJIP transients were analyzed using JIP-test, and the JIP-test is a multiparametric analysis of the OJIP transients, which is based on the theory of energy fluxes in bio-membranes (Strasser, 1981; Strasser and Strasser, 1995). From OJIP transient, the extracted parameters (*F*_20 µs_, *F*_300 µs_, *F*_2 ms_, *F*_30 ms_ etc.) led to the calculation and derivation of a range of new parameters according to previous authors (**Table S1**; Yusuf et al., 2010).

### Leaf anatomical characteristics under different light regimes

After photosynthetic parameters measurement, leaf sections of 1.00 × 1.00 cm were also cut from the middle of fully expanded function leaves (avoiding midribs). Leaves were cleaned by sterilizing water and stored in the FAA fixative. Leaf tissues were dyed by hematoxylin staining method and fixed with paraffin before observed (Xiong et al., 2017; Chang et al., 2023). The tissue sections were observed under electron microscope and analyzed through separately quantifying variables in the visible field using Case Viewer software.

### Determination of antioxidant enzyme activities

Leaf was homogenized on ice with a mortar and pestle in a 0.1 M potassium phosphate buffer (pH 7.0). The homogenate was centrifuged at 12000 *g* for 15 min at 4°C. The supernatant was used immediately for enzyme assays (Wang et al., 2009). The activity of superoxide dismutase (SOD) was measured according to a method using xanthine, xanthine oxidase, and cytochrome *c* (Giannopolitis and Ries, 1977). The activity of peroxidase (POD) was assayed according to the method described by Zhang et al. (2005), using pyrogallol as a substrate. Catalase (CAT) activity was assayed according to the method described by Aebi (1984), by measuring the decrease at 240 nm for 1 min, due to H_2_O_2_ consumption.

### Statistical analyses

SPSS 20.0 software (Chicago, IL, USA) was used to statistical analysis. The variables were means ± standard deviation (SD) (*n* = 7). Significant differences are indicated by letters (One-way ANOVA; *P* < 0.05). Graphing was made by SigmaPlot 10.0 (Systat Software Inc, San Jose) and GraphPad Prism 8.0 (GraphPad Inc, USA) software.

## Results

### Response of the Chl contents to light regimes

**Figure 1:**
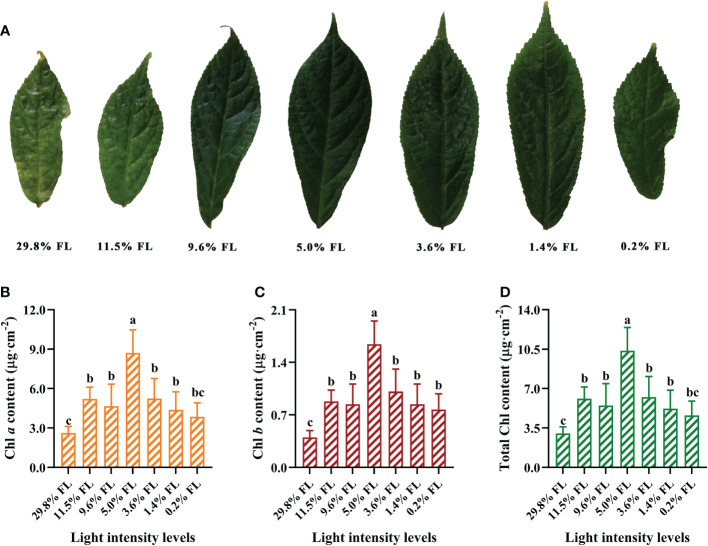
The effect of light regimes on leaf phenotypes **(A)**, cited from our research group (Zhang et al., 2021), chlorophyll *a* (Chl *a*) content (μg·cm^-2^, **B**), chlorophyll *b* (Chl *b*) content (μg·cm^-2^, **C**) and total Chl content (μg·cm^-2^, **D**). Values for each point were means ± SD (*n* = 7). Letters indicate significant differences at *P < 0.05* according to Duncan’s multiple range tests.

Leaves were significantly smaller and yellowish in *P. notoginseng* under high light; moderate-light-grown leaves were dark-green (**Figure 1A**). The content of Chl *a*, Chl *b*, total Chl increased first and then decreased with the increase of growth irradiance (**Figures 1B–D**). The maximum values of photosynthetic pigments were recorded in 5.0% FL-grown *P. notoginseng* (**Figure 1**; as reflected by Chl *a*, Chl *b*, total Chl content). Chl *a*, Chl *b*, total Chl contents were lowest in *P. notoginseng* under 29.8% FL (**Figures 1B–D**).

### The effect of grown irradiance on gas exchange

**Figure 2:**
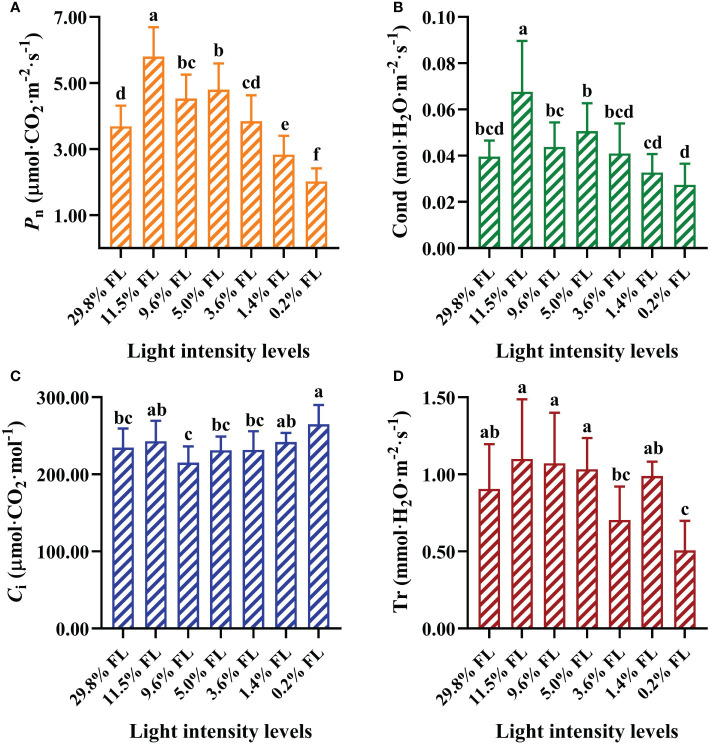
Effects of long-term light treatments on gas exchange parameters in *Panax notoginseng* leaves. **(A)** Net photosynthesis rate (*P*_n_, μmol·CO_2_·m^-2^·s^-1^). **(B)** Stomatal conductance (Cond, mol·H_2_O·m^-2^·s^-1^). **(C)** Intercellular CO_2_ concentration (*C*_i_, μmol·CO_2_·mol^-1^). **(D)** Transpiration rate (Tr, mmol·H_2_O·m^-2^·s^-1^). Values for each point were means ± SD (*n* = 7). Letters indicate significant differences at *P < 0.05* according to Duncan’s multiple range tests.

*P*_n_ and Cond were significantly enhanced in 11.5% FL-grown plants compared with other treatments (**Figures 2A, B**). Compared with 11.5% FL-grown *P. notoginseng*, *P*_n_ were decreased 36.55% and 65.17% in 29.8% FL- and 0.2% FL-grown plants, respectively (**Figure 2A**). The maximum and minimum values of *C*_i_ were recorded in 0.2% FL- and 9.6% FL-grown plants, respectively (**Figure 2C**). The minimum values of *P*_n_, Cond, and Tr were obtained in *P. notoginseng* under 0.2% FL condition (**Figures 2A, B, D**).

### The effect of growth irradiance on leaf anatomical characteristics

The thickness of the upper epidermis, palisade tissue, and lower epidermis were declined with increasing growth irradiance (**Table 1**, **Figure S2**). 29.8% FL-grown leaves were dramatically increased in the thickness of the upper epidermis, palisade tissue, and spongy tissue (**Table 1**). The thickness of the lower epidermis was greatest in *P. notoginseng* grown under 29.8% and 11.5% FL condition (**Table 1**). These differences were not significant for the upper epidermis thickness in the range 3.6% to 11.5% FL (**Table 1**). The palisade/spongy increased first and then decreased with the increase of growth irradiance, and the maximum values of palisade/spongy were recorded in 5.0% FL-grown plants (**Table 1**).

**Table 1:** Effects of light regimes on the leaf anatomy in a shade tolerant plant *Panax notoginseng*.

Variables	Growth irradiance (% of full sunlight, % FL)						
	29.8% FL	11.5% FL	9.6% FL	5.0% FL	3.6% FL	1.4% FL	0.2% FL
Upper epidermis (μm)	16.09 ± 3.45 a	12.74 ± 2.27 b	12.36 ± 1.80 b	12.21 ± 2.33 b	12.16 ± 2.32 b	8.33 ± 1.65 c	8.13 ± 2.29 c
Palisade tissue (μm)	36.26 ± 5.55 a	28.37 ± 6.15 b	29.59 ± 4.47 b	29.06 ± 6.17 b	20.48 ± 3.26 c	16.71 ± 2.40 d	13.73 ± 3.08 e
Spongy tissue (μm)	58.59 ± 15.76 a	39.17 ± 10.32 b	35.59 ± 5.62 bc	30.52 ± 5.88 cd	37.32 ± 6.41 b	29.04 ± 5.49 d	28.4 ± 6.71 d
Lower epidermis (μm)	12.91 ± 2.38 a	13.53 ± 2.05 a	11.49 ± 2.36 b	11.28 ± 1.84 bc	9.96 ± 2.01 c	7.80 ± 2.23 d	7.37 ± 2.63 d
Palisade/Spongy	0.65 ± 0.16 cd	0.77 ± 0.25 bc	0.86 ± 0.21 bc	1.00 ± 0.38 a	0.56 ± 0.11 de	0.60 ± 0.15 de	0.50 ± 0.11 e

Values are means ± SD. (n = 7). Different letters among light regimes indicate significant difference (P < 0.05).

### Response of the photosystem activity to light regimes

**Figure 3:**
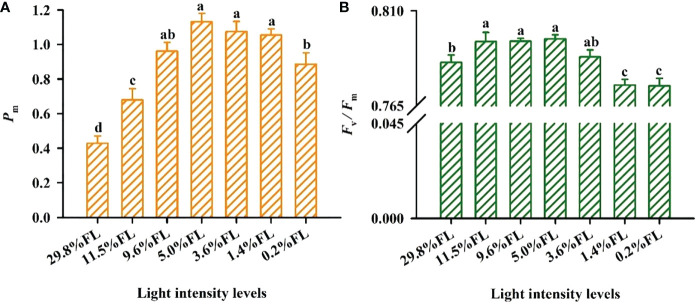
The effect of light regimes on PSI and PSII activity of *Panax notoginseng.* **(A)** *P*_m_ is the maximum photo-oxidation ${\text{P}}_{700}^{+}$ . **(B)** *F*_v_/*F*_m_ is the maximum efficiency of PSII photochemistry. Values for each point were means ± SD (*n* = 7). Letters indicate significant differences at *P < 0.05* according to Duncan’s multiple range tests.

Growth irradiance significantly influenced PSI and PSII activity in the leaf (**Figure 3**). The minimum values of *F*_v_/*F*_m_ were showed in *P. notoginseng* grown under long-term low light (1.4% FL, 0.2% FL) (**Figure 3B**), and *P*_m_ in high-light-grown plants were lower (29.8% FL, 11.5% FL) (**Figure 3A**). The difference between moderate- and low-light-grown plants in *P*_m_ was only marginal (**Figure 3A**), but *P*_m_ was highest in *P. notoginseng* grown under 5.0% FL (**Figure 3A**).

### Response of the photosynthetic electron transport to light regimes

**Figure 4:**
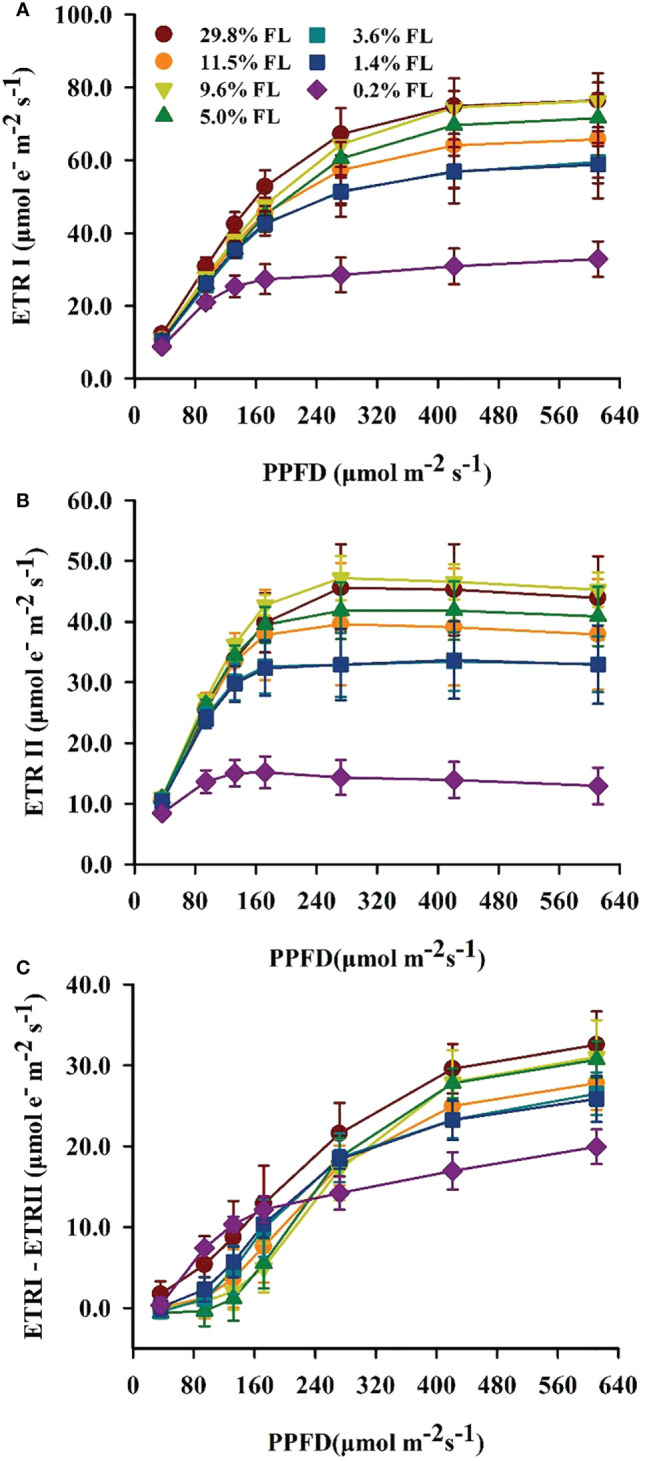
Characteristics of electron transport between PSII and PSI in 1eaves of *P. notoginseng* grown under different light levels. **(A)** Response of electron transport rate of PSI (ETRI, μmol·e^-^·m^-2^·s^-1^) to photosynthetic photon flux density (PPFD, μmol·m^-2^·s^-1^). **(B)** Response of electro transport rate of PSII (ETRII, μmol·e^-^·m^-2^·s^-1^) to PPFD. **(C)** Response of cyclic electron flow around PSI (ETRI - ETRII, μmol·e^-^·m^-2^·s^-1^) to PPFD. Values for each point were means ± SD (*n* = 7).

ETRI, ETRII and ETRI - ETRII were raised with increasing PPFD (**Figure 4**). ETRI and ETRII were significantly greater in 29.8% FL- and 9.6% FL-grown plants compared with other individuals (**Figures 4A, B**). ETRI and ETRII were significantly reduced in low-light-grown plants (0.2% FL; **Figures 4A, B**). When PPFD was lower than 200 μmol·m^-2^·s^-1^, the maximum values of ETRI - ETRII were obtained in 0.2% FL and 29.8% FL *P. notoginseng* (**Figure 4C**). When plants were exposed to higher PPFD, the maximum values of ETRI - ETRII were recorded in 29.8% FL individuals, but the ETRI - ETRII were declined in low-light-grown *P. notoginseng* (0.2% FL, **Figure 4C**).

### Response of the light energy partitioning to growth irradiance

**Figure 5:**
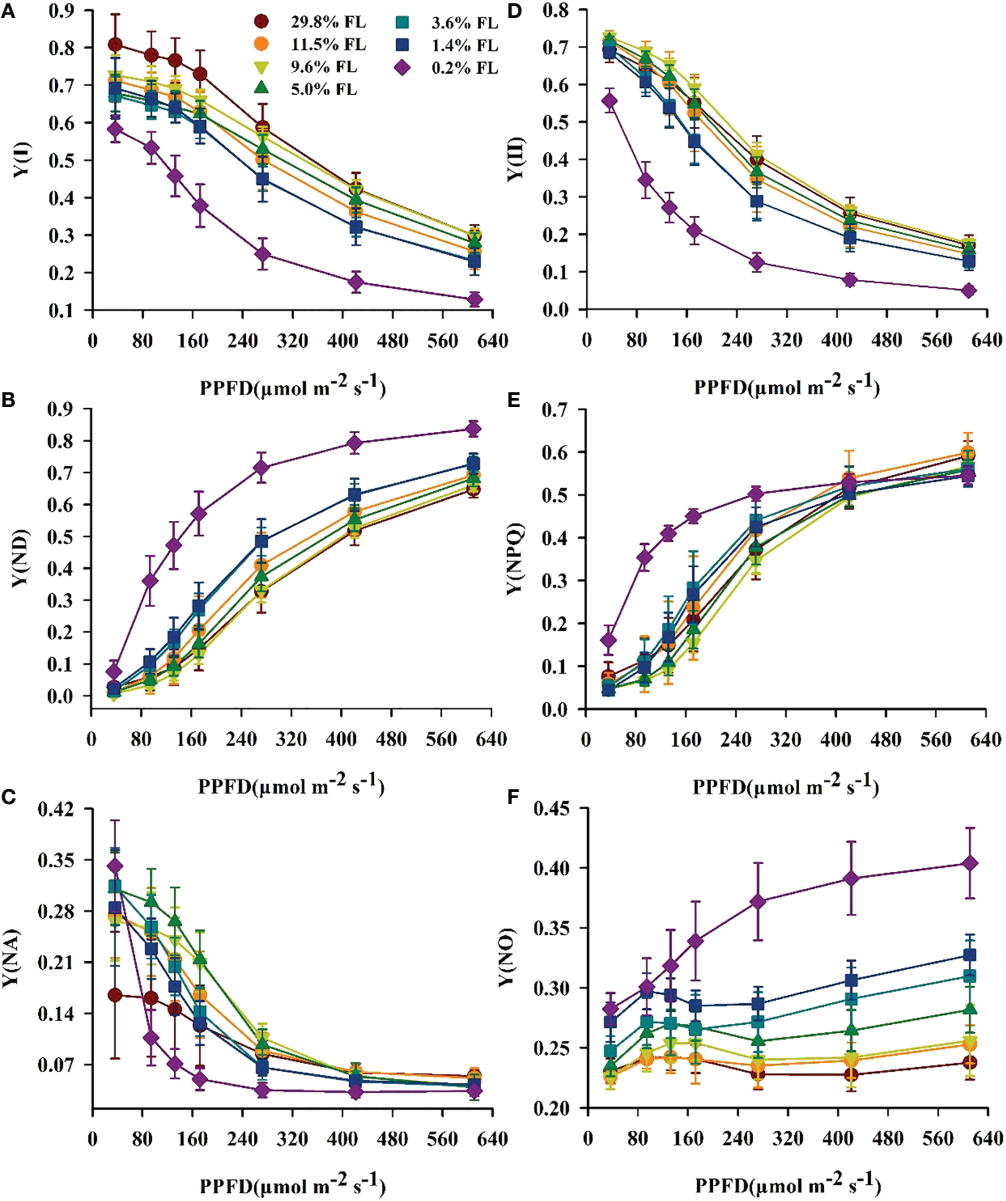
The effect of light regimes on light energy allocation in *P. notoginseng*. **(A)** Y(I) is the quantum yield of PSI. **(B)** Y(ND) is the donor side limitation of PSI. **(C)** Y(NA) is the acceptor side limitation of PSI. **(D)** Y(II) is the efficient quantum yield of PSII. **(E)** Y(NPQ) is the yield of regulated energy dissipation of PSII. **(F)** Y(NO) is the yield of non-regulated energy dissipation of PSII. Values for each point were means ± SD (*n* = 7).

**Figure 6:**
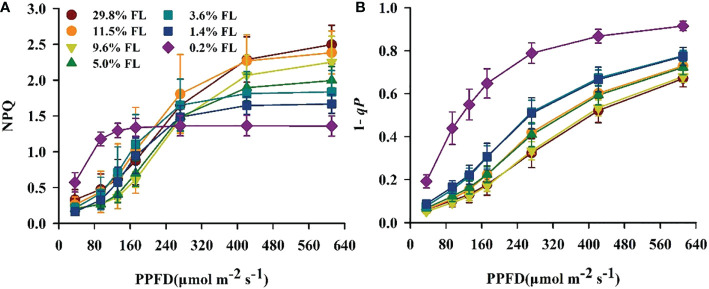
Changes of fluorescence characteristics in the light response process in *P. notoginseng* under different levels of light. **(A)** NPQ is the non-photochemical quenching of PSII. **(B)** 1-*qP* is the light response changes in the redox poise of the primary electron acceptor of PSII. Values for each point were means ± SD (*n* = 7).

The minimum values of Y(I) were shown in the 0.2% FL individuals (**Figure 5A**), and Y(ND) in low-light-grown individuals was greatest (**Figure 5B**). The opposite of Y(ND), Y(NA) was increased when PPFD is lower than 272 μmol·m^-2^·s^-1^ in plants grown under moderate shading environments (**Figure 5C**). There was no significant difference in Y(NA) when PPFD is more than 272 μmol·m^-2^·s^-1^. Compared with PSI, the lowest values of Y(II) were always observed in low-light-grown *P. notoginseng* (**Figure 5D**), and Y(NPQ) was highest in 0.2% FL plants (**Figure 5E**). Y(NO) was rapidly increased when PPFD is higher than 272 μmol·m^-2^·s^-1^ (**Figure 5F**), and the Y(NO) were increased in low-light-grown plants (**Figure 5F**). NPQ and 1-*qP* increased with increasing PPFD (**Figure 6**). NPQ was increased in *P. notoginseng* were exposed to high light (29.8% FL, 11.5% FL; **Figure 6A**), and 1-*qP* in 0.2% FL plants were highest (**Figure 6B**).

### Response of the cycle electron flow around PSI to light stress

**Figure 7:**
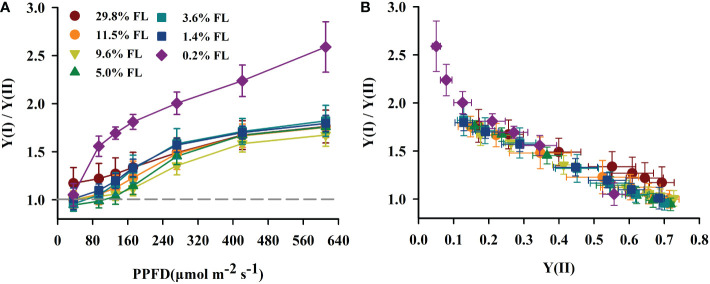
The effect of light regimes on cyclic electro transport in *P. notoginseng*. **(A)** Light response changes in Y(I)/Y(II) for leaves of *P. notoginseng* grown under different light regimes. Above the gray line represents the start of cyclic electron transport being excited. **(B)** Relation between Y(I)/Y(II) and Y(II) (line electro transport) for leaves of *P. notoginseng* grown under different light regimes. Values for each point were means ± SD (*n* = 7).

**Figure 8:**
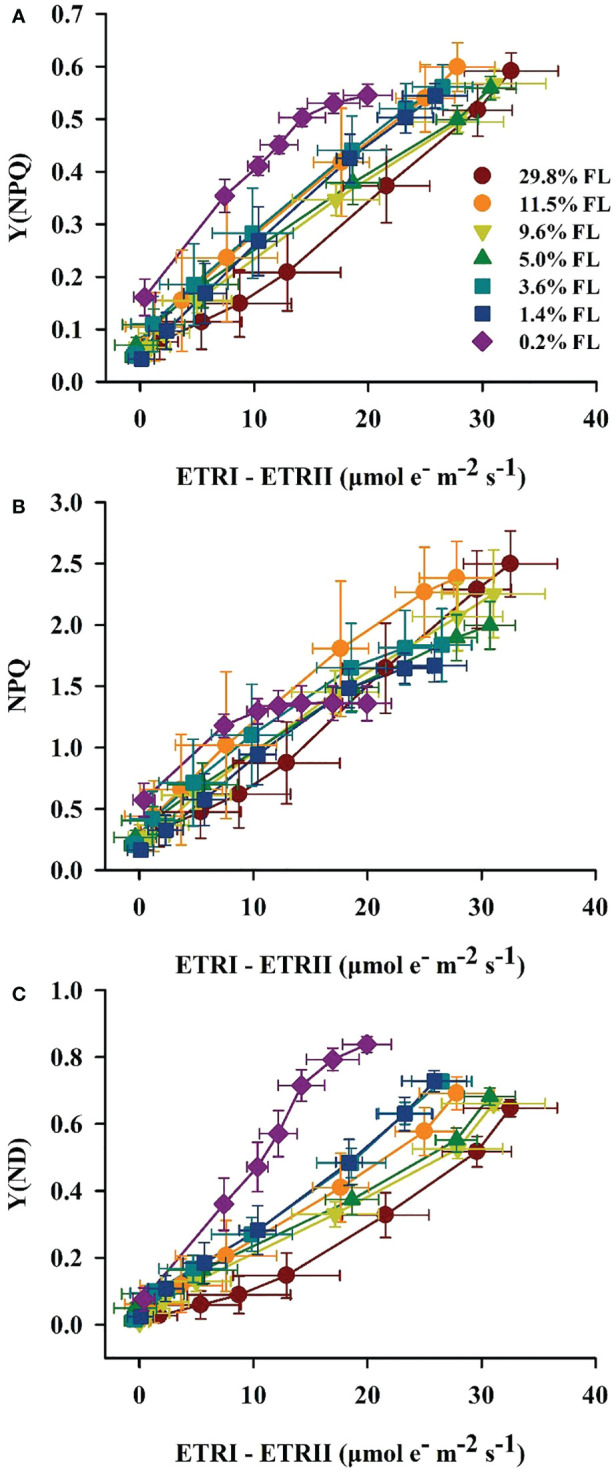
Relation between ETRI - ETRII and Y(NPQ) **(A)**, NPQ **(B)**, Y(ND) **(C)** for leaves of *P. notoginseng* grown under different light regimes. Values for each point were means ± SD (*n* = 7).

The quantum yield of cyclic electron flow around PSI [Y(I)/Y(II)] increased with increasing PPFD (**Figure 7A**). Y(I)/Y(II) was activated earlier when PPFD was higher than 36 μmol·m^-2^·s^-1^ in *P. notoginseng* under light stress (29.8% FL, 0.2% FL; **Figure 7A**). Y(I)/Y(II) was inversely correlated with Y(II) (**Figures 5D**, **7B**), and the greatest values were shown in 0.2% FL individuals (**Figure 7B**). As showed in **Figure 8**, Y(NPQ), NPQ and Y(ND) were positively correlated with ETRI - ETRII (**Figure 8**). Y(NPQ), NPQ and Y(ND) were greatest in the 0.2% FL individuals when ETRI - ETRII is lower (**Figure 8**). Y(NPQ), NPQ and Y(ND) were increased in the high-light-grown plants when ETRI - ETRII was greater (**Figure 8**).

### Changes in activities of antioxidant enzymes

**Figure 9:**
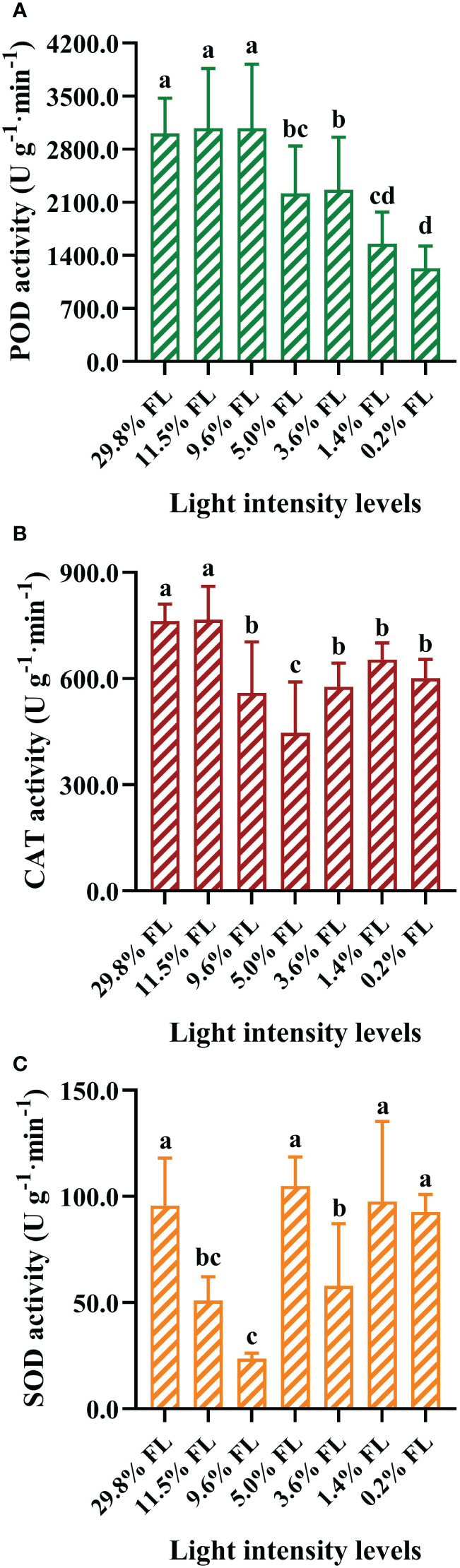
The effects of light stress on the antioxidant activities ofperoxidase (POD), catalase (CAT) and superoxide dismutase (SOD) in the leaves of *P. notoginseng.* **(A)** POD activity (U g^-1^·min^-1^). **(B)** CAT activity (U g^-1^·min^-1^). **(C)** SOD activity (U g^-1^·min^-1^). Values for each point were means ± SD (*n* = 7). Letters indicate significant differences at *P < 0.05* according to Duncan’s multiple range tests.

POD activity was greater in *P. notoginseng* grown under 29.8%, 11.5%, and 9.6% FL condition (**Figure 9A**, *P* < 0.05). The POD activity was declined with decreasing growth irradiance (**Figure 9A**), and the minimum values of POD activity was obtained in 0.2% FL-grown *P. notoginseng* (**Figure 9A**). CAT activity was significantly increased in high-light-grown plant (29.8% FL, 11.5% FL; **Figure 9B**). CAT activity was lowest in 5.0% FL-grown plants (**Figure 9B**). SOD activity was reduced with decreasing grown irradiance in the range 29.8% to 9.6% FL (**Figure 9C**). SOD activity was significantly decreased in 3.6% FL-grown plants compared with 5.0%, 1.4% and 0.2% FL treatments (**Figure 9C**, *P* < 0.05).

### Response of the OJIP kinetic curve to light regimes

**Figure 10:**
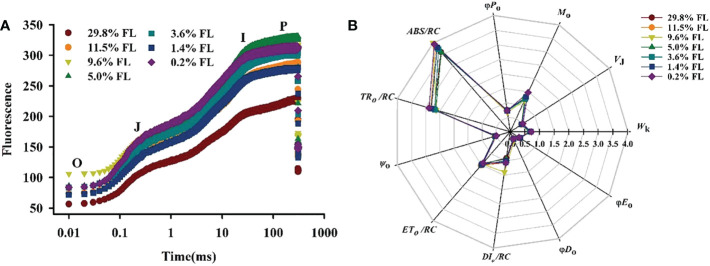
Effects of light regimes on chlorophyll fluorescence transients of *P. notoginseng.* **(A)** O, J, I and P phase represent the fluorescence at T=20 μs, 2 ms, 30 ms and 300 ms, respectively. **(B)** A radar plot of JIP parameters in P. notoginseng leaves grown under different light regimes. *ABS/RC* is the absorption flux per reaction center of PSII; *TR_o_/RC* is the captured light energy used to restore *q*_A_; *ET_o_/RC* is the captured light energy used for electron transfer per unit area; *DI_o_/RC* is the energy dissipated per unit reaction;*Ψ*_o_ is the probability that a trapped exciton moves an electron into the electron transport chain beyond ${\text{Q}}_{A}^{-}$ (at *t*=0); *M*_o_is the approximated initial slope of the fluorescence transient; *V*_J_ is the relative variable fluorescence intensity at the J-step; *W*_k_ is the K phase in O-J-I-P chlorophyll fluorescence induction curves; *φD_o_* is the quantum yield for thermal dissipation; *φE_o_* is the quantum yield for electron transport (*t* = 0); *φP_o_* is the maximum quantum yield for primary photochemistry (*t* = 0). Values for each point were means (*n* = 7).

**Figure 11:**
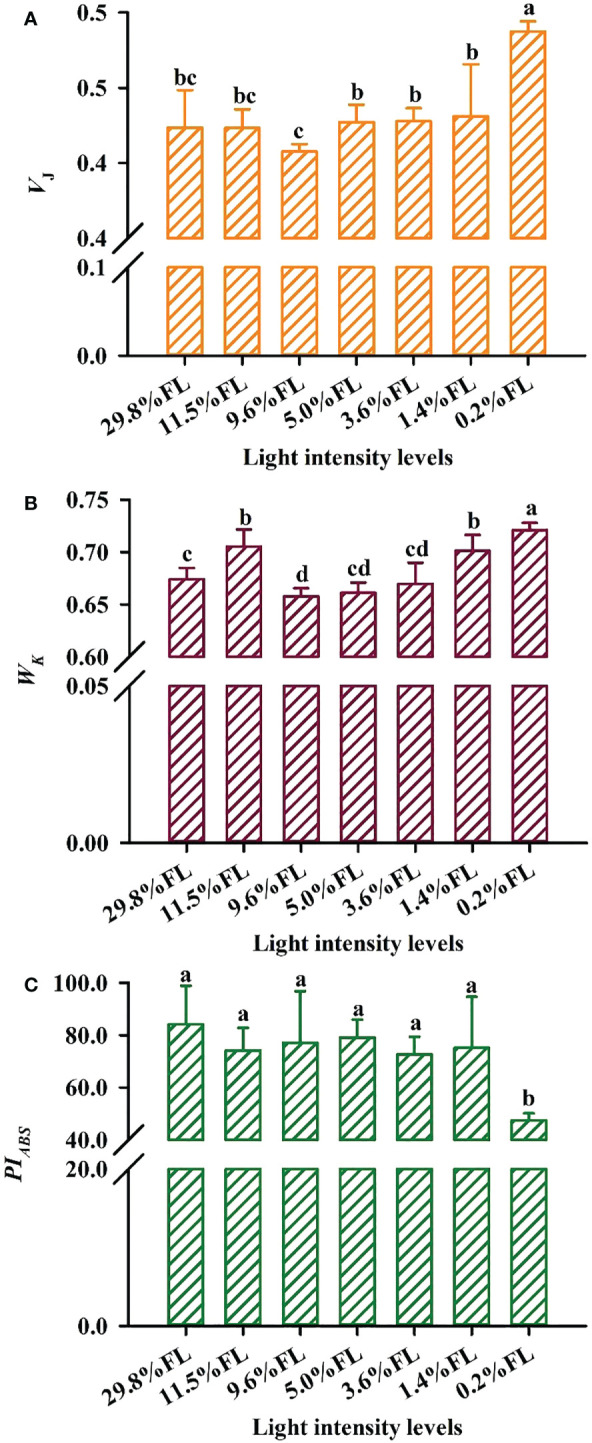
Effect of light regimes on the *V*_J_,*W*_k_ and *PI_ABS_* of *P. notoginseng* leaves. **(A)** *V*_J_ is the relative variable fluorescence intensity at the J-step; **(B)** *W*_k_ is the K phase in O-J-I-P chlorophyll fluorescence induction curves. **(C)** *PI_ABS_* is the performance index on absorption basis. Values for each point were means ± SD (*n* = 7). Letters indicate significant differences at *P < 0.05* according to Duncan’s multiple range tests.

The OJIP kinetic curve showed an “S”-shaped in all light regimes (**Figure 10A**). The lower fluorescence values were shown in high-light-grown individuals, *F*_o_≌*F*_20 μs_ (O phase) was greater in the 9.6% FL individuals, and the maximum values of *F*_M_=*F*_P_=*F*_300 ms_ (P phase) were recorded in the 5.0% FL individuals (**Figure 10A**). *W*_k_ was lower in moderate-light-grown plants (9.6% FL, 5.0% FL, 3.6% FL; **Figure 11B**), and the maximum values of *W*_k_ were recorded in 0.2% FL individuals (**Figure 11B**).

In the JIP-test parameters, change in *M*_o_, *V*_J_ and *ψ*_o_ can reflect activity of PSII acceptor sides (Force et al., 2003). Changes of *M*_o_ and *V*_J_ are similar (**Figures 10B**, **11A**), and *M*_o_ and *V*_J_ were greater in low-light-grown plants (0.2% FL, **Figures 10B**, **11A**). *ψ*_o_ was significantly lower in 0.2% FL plants than in other light regimes plants (**Figure 10B**). Compared with *F*_v_/*F*_m_, *PI_ABS_* could more sensitively reflect the activity of PSII acceptor sides (Crafts-Brandner and Salvucci, 2002). The minimum values of *PI_ABS_* were surveyed in 0.2% FL individuals (**Figure 11C**), and there were not significantly different in other light regimes (**Figure 11C**). *DI_o_/RC* and *ABS/RC* were highest in the 9.6% FL plants (**Figure 10B**), and *ET_o_/RC* were higher in low-light-grown individuals (0.2% FL; **Figure 10B**). *ABS/RC* and *TR_o_/RC* were increased when the growth irradiance is lower than 5.0% FL (**Figure 10B**).

### Phenotypic plasticity index analysis for Chl fluorescence-related parameters

**Figure 12:**
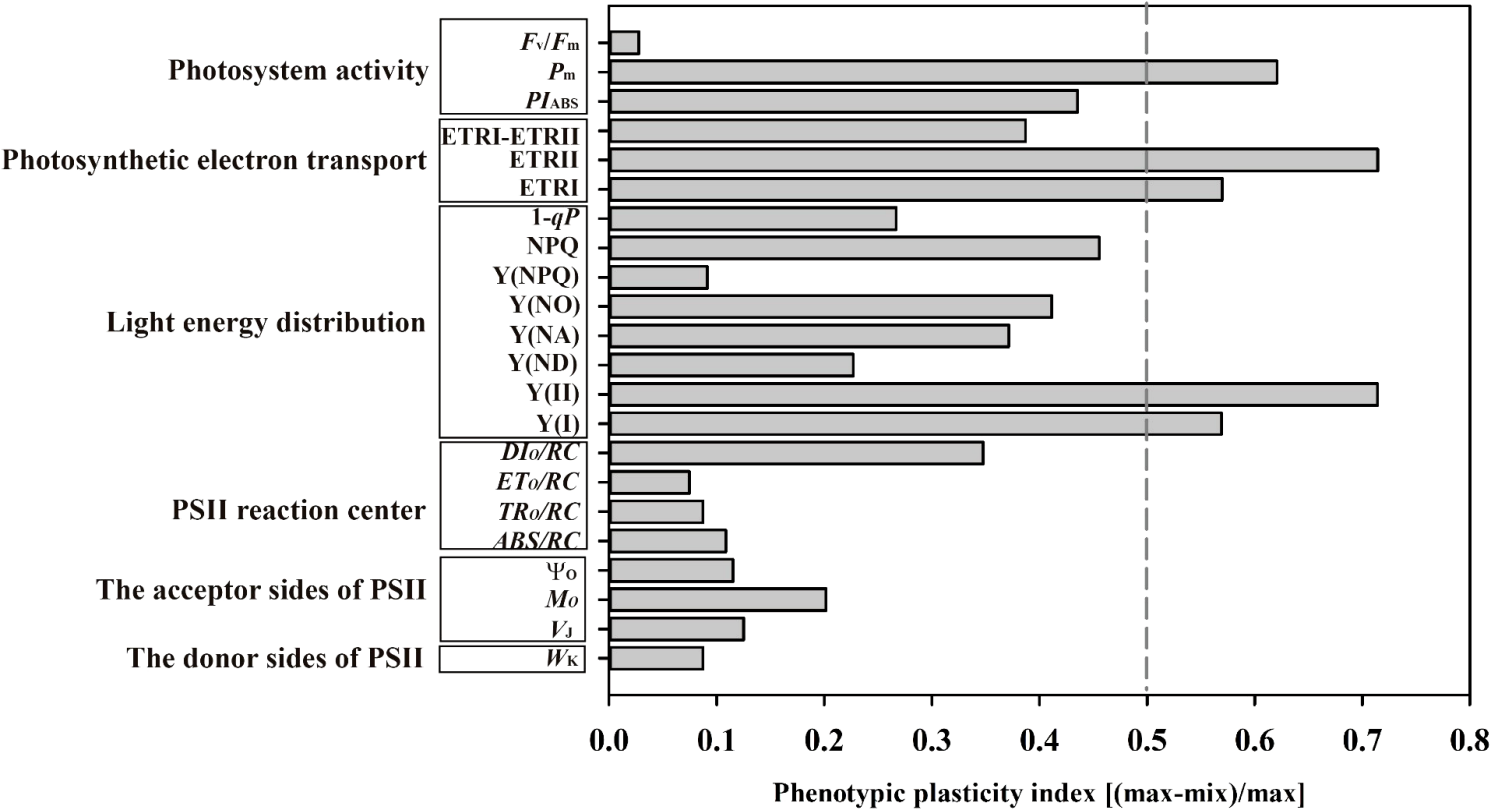
Phenotypic plasticity index of the twenty-two chlorophyll fluorescence variables of photosystem activity, photosynthetic electron transport, light energy distribution, PSII reaction center, the acceptor sides and donor sides of PSII. Means were calculated for seven individuals for each light treatment.

The plasticity index of *P*_m_ was much greater than that of *F*_v_/*F*_m_ among the photosystem activity variables (**Figure 12**); The higher plasticity index values of ETRI, ETRII, Y(II) and Y(I) were shown among photosynthetic electron transport and light energy distribution (**Figure 12**). The plasticity indices of *M*_o_ were largest among PSII receptor side parameters (**Figure 12**). Noteworthy, the plasticity indices of *P*_m_, ETRII, ETRI, Y(II) and Y(I) exceeded 0.5, and the lowest plasticity indices values of *F*_v_/*F*_m_, Y(NPQ), *ET_o_/RC* and *W*_k_ (**Figure 12**).

## Discussion

### Light-driven changes in photosynthesis is in part explained by leaf anatomy

Photosynthetic capacity is at least in part determined by leaf anatomy and *P*_n_ is limited by the rate of CO_2_ diffusion from the atmosphere to the chloroplast (Gratani and Bombelli, 2000). The reduction of palisade tissue thickness increases the density of chloroplast distribution and enchants light-receiving area and light capture capability, thus improving photosynthetic capacity in shade -tolerant species (e.g., *Phoebe bournei*, *Cyclobalanopsis gilva*, *Zelkova serrata*, *Cinnamomum camphora*; Xue, 2020). Thicker upper epidermis protects mesophyll tissue from damage in high-light-grown *Acer rybrum* (Goulet and Pierre, 1986). The thickness of palisade tissue was declined with increasing growth irradiance, and 29.8% FL-grown leaves were dramatically increased in the thickness of the upper epidermis (**Table 1**, **Figure S2**). These results imply that *P. notoginseng* leaves made favorable adaption to high and low light, respectively. Correspondingly, the increase of upper epidermis, palisade tissue, and lower epidermis would reduce liquid phase diffusion of CO_2_ in mesophyll cells (**Table 1**), this might partly explain the fact that a significant decline in *P*_n_ was observed in the high-light-grown plants (**Figure 2**), as has also been observed in Zhang et al. (2020). Meanwhile, low-light-grown leaves were declined in Tr and Cond, and *C*_i_ and *P*_n_ had opposite trends (**Figure 2**). These results imply that the decline of photosynthetic rate in low-light-grown *P. notoginseng* was mainly caused by non-stomatal limitation factors, and this is consistent with the results reported by Rylski and Spigelman (1986). Thus, light-driven changes in *P*_n_ are in part explained by leaf anatomy.

### Low light stress exacerbates photoinhibition to PSII in the shade-tolerant species

It has commonly accepted that the primary sites of photoinhibition are PSI and PSII (Gerganova et al., 2016). The PSI and PSII photoinhibition is characterized by a significant decrease in *P*_m_ and *F*_v_/*F*_m_, respectively (Demmig-Adams and Adams, 1992). PSII activity is inhibited under high light, but PSI activity remains stable, and this has been confirmed in *Solanum lycopersicum* and *Arabidopsis thaliana* (Gerganova et al., 2019; Chen et al., 2020). *F*_v_/*F*_m_ was greatly reduced in 1.4% FL- and 0.2% FL-grown plants (**Figure 3B**), but PSI activity was relatively increased in low-light-grown plants (**Figure 3A**). This is inconsistent with the results reported that inhibition of the activity of PSII under strong light is referred to as photoinhibition (Murata et al., 2007). This may be due to the different light demands of the study species (as reflected by *P. notoginseng* is a typically shade-tolerant species). These results imply that the degree of PSII photoinhibition is significantly affected by long-term low light stress, as confirmed in the shade-tolerant species *P. henryi* treated by short-term low light (Huang et al., 2016b). Meanwhile, the degree of inhibition of *P*_n_ under 0.2% FL was greater than that of 29.8% FL (**Figure 2A**), it implied that *P. notoginseng* are more sensitive to long-term low light compared to high light. Furthermore, compared with *F*_v_/*F*_m_, *PI_ABS_* could more sensitively reflect the activity of PSII (Crafts-Brandner and Salvucci, 2002; Li et al., 2009b). *PI_ABS_* in 0.2% FL plants was significantly lowest than other counterparts (**Figure 11C**). Obviously, PSII was more sensitive to low light stress compared with PSI. Therefore, long-term low light stress exacerbates the photoinhibition to PSII in the shade-tolerant species.

### PSI photoinhibition is a fundamental reason for the sensitivity of the shade-tolerant plants to high light

PSI activity is slow to recover from photoinhibition compared with the recovery of PSII activity (Zhang and Scheller, 2001; Zhou et al., 2019). PSI photoinhibition mainly occurs in plants grown under high light and chilling temperatures condition (Zhang and Scheller, 2001), as has been recorded in the shade-tolerant plants *P. rubra*, *P. henryi* and *Nephrolepis falciformis* (Huang et al., 2015; Huang et al., 2017; Huang et al., 2018b). *P*_m_ in 29.8% FL plants was greatly reduced by 51.57% in relative to 0.2% FL counterparts (**Figure 3A**), and PSI activity is significantly reduced in high-light-grown plants. The excess electrons on PSI acceptor side induce the formation of superoxide anion radicals and the reduction of the iron-sulfur center in PSI, which leads to photoinhibition to PSI (Sonoike, 2011). Y(NA) in 29.8% FL individuals was significantly higher than 0.2% FL individuals (**Figure 5C**), implying that the occurrence of PSI photoinhibition in high-light-grown *P. notoginseng* might is due to the excess accumulation of superoxide anion radicals on the PSI acceptor side as has been proposed by Kim et al. (2005). PSI is sensitive in high-light-grown *P. notoginseng*. On the other hand, the degree of PSI photoinhibition is greater than that of PSII photoinhibition in high-light-grown individuals (**Figure 3**), and the plasticity index of *P*_m_ was larger than that of *F*_v_/*F*_m_ (**Figure 12**). PSI photoinhibition is the basis for the sensitivity of shade-tolerant plants *P. rubra* to high light condition (Huang et al., 2015). Thus, PSI photoinhibition might be a vital reason for explaining why the shade-tolerant plants *P. notoginseng* cannot grow under high light.

### Enhanced photosynthetic electron transport and moderate PSII photoinhibition in high-light-grown plants

On the condition of excess light, the utilization and dissipation of light are increased to protect PSII and PSI against photoinhibition (Zhang et al., 2015; Bascuñán-Godoy et al., 2018). Higher NPQ dissipates excess energy as heat in order to prevent damage to PSII of high-light-grown *A. thaliana* and *Chromera velia* (Belgio et al., 2018; Howard et al., 2019). 29.8% FL-grown plants possessed a high NPQ (**Figure 6**). These results imply that excess light energy could be effectively dissipated in the form of heat photochemistry in high-light-grown plants. Thus, high-light-grown plants show greater photochemical efficiency and photoprotective capacity, contributed by higher Y(II) and NPQ (**Figures 5D**, **6A**, **8**), while the NPQ of shade plants is more sensitive to changes in high light. This is consistent with the results reported by Ishida et al. (2014) that a larger proportion of Y(II) and Y(NPQ) has been observed in high-light-grown *O. sativa.* Moreover, the utilization of excess light is increased by increasing electron transport and photochemistry in high-light-grown (Genty and Harbinson, 1996). Y(I), Y(II), ETRI, ETRII and NPQ were increased in the 29.80% FL individuals (**Figures 4**, **5**, **6A**); and the plasticity indices of ETRII, ETRI, Y(II) and Y(I) all exceeded 0.5 (**Figure 12**). These results imply that excess light energy could be effectively dissipated in the form of heat or photochemistry in high-light-grown plants. However, excess light energy could not be effectively dissipated in time, which accumulates ROS (Zhou et al., 2019). Plants up-regulate the antioxidant enzyme system to scavengethe ROS under stress (Li et al., 2009). The activities of SOD, POD and CAT showed different degrees of changes in high-light-grown *P. notoginseng* (**Figure 9**). This is consistent with the results reported by Zhang et al. (2022) that the activation of SOD and POD could avoid photooxidative damage in *Pyropia haitanensis* grown under high light condition. Overall, high-light-grown *P. notoginseng* had stronger capability of scavenging ROS and non-photochemical quenching. Moreover, light capture capability was decreased by inhabiting Chl content (as reflected by Chl *a*, Chl *b*, and total Chl content) in 29.80% FL-grown *P. notoginseng* (**Figures 1B-D**), as has been confirmed by Sato et al. (2015) in *A. thaliana* grown under high light stress. The degree of PSI photoinhibition is higher than that of PSII photoinhibition in high-light-grown *P. notoginseng* (**Figure 3**). PSI photoinhibition in *P. notoginseng* grown under high light condition was primarily caused by the excess electron transport from PSII to PSI (Huang et al., 2015). PSI activity is protected against photodamage in *pgr5* mutants of *A. thaliana* upon moderate PSII photoinhibition, due to the depression of electron flow from PSII to PSI (Tikkanen et al., 2014). Moderate photoinhibition of PSII is a protective response (Huang et al., 2016a; Huang et al., 2018a). *F*_v_/*F*_m_, *Ψ*_o_, *W*_K_ and *V*_J_ were relatively stable when *P. notoginseng* were exposed to high light (**Figures 3B**, **10B**, **11A, B**), as has been confirmed by Thachle et al. (2007) in *Graptophyllum reticulatum*. These results imply that moderate photoinhibition of PSII occurs in high-light-grown *P. notoginseng*. Therefore, the enhanced photosynthetic electron transport and moderate PSII photoinhibition of *P. notoginseng* under high light condition were presented as photoprotection strategies.

### Low light stress damages the acceptor side of PSII

The enhanced absorption and utilization of light energy is a predominated strategy for plants to adapt to low light (Lei et al., 1996; Ruberti et al., 2012), and this has been confirmed in the shade-tolerant species *Paeonia veitchii*, *Paeonia intermedia* and *Paeonia anomala* grown under low light (Wan et al., 2020). *ABS/RC*, *TR_o_/RC*, 1-*qP*, and *M*_o_ were enhanced in 0.2% FL-grown *P. notoginseng* (**Figures 6B**, **10B**). The capture and absorption of light energy were improved by the increased active reaction centers per unit area in *P. notoginseng* grown under low light. Additionally, antenna sizes are increased by enhancing Chl *b* and LHCII levels in low-light-grown *A. thaliana*, resulting in higher light capture capability (Sato et al., 2015). The previous observation is consistent with present results that the maximum values of Chl *b* content were recorded in 5.0% FL-grown *P. notoginseng* (**Figure 1C**). These results imply that light capture capability is enhanced by increasing antenna size in *P. notoginseng* grown under low-light stress.

It has commonly accepted that the state transition is a photoprotective mechanism that improves the utilization of plant light energy by balancing the excitation energy of PSI and PSII (Bailey and Grossman, 2008; Khuong et al., 2019). In the present study, the maximum values of 1-*qP* were recorded in 0.2% FL plants (**Figure 6B**). The maintenance of state 1 of *P. notoginseng* at 0.2% FL may be due to the strong PSII excitation, resulting in high excitation pressure on PSII (Tikkanen et al., 2006). These results imply that PSII reaction centers are inactivated in plants grown under low light, as has been confirmed by Chen and Xu (2006). However, the imbalance between the absorption and utilization of light energy could cause a damage to photosynthetic apparatus (Zavafer et al., 2019; Kodru et al., 2020). Y(II), Y(I), NPQ, *φD_o_* and *F*_v_/*F*_m_ were decreased in the 0.2% FL individuals, but Y(NO) was increased (**Figures 3B**, **5A,D,F**, **6A**, **10B**), suggesting that excess light energy could not be effectively dissipated in the form of thermal in low-light-grown individuals, and it probably lead to the reduction in PSII activity and the damage to PSII. On the other hand, plants would use light energy through photosynthetic electron transport to protect photosynthetic apparatus, and this has been confirmed in the light-demanding species *Shorea leprosula* and *Cerasus cerasoides* grown under light stress (Scholes et al., 1996; Yang et al., 2019b). ETRI, ETRII, ETRI - ETRII, *ET_o_/RC* and *F*_v_/*F*_m_ were reduced in low-light-grown *P. notoginseng* (0.2% FL or 1.4% FL; **Figures 3B**, **4**, **10B**). Low-light-grown *P. notoginseng* cannot increase the utilization of light energy by enhancing electron transport. The decline in PSII activity result in the inhibition to electron transport in low-light-grown *P. notoginseng* (**Figures 3B**, **4**). This is consistent with the results reported by Huang et al. (2018a) that the decline in electron transport under low light is induced by a decline in PSII activity in *P. notoginseng.* The imbalance between PSI and PSII leads to reduced electron transport (Wen et al., 2005; Sonoike, 2011; Oguchi et al., 2021). The previous observation is consistent with present results that the lower value of ETRI, ETRII and *ψ*_o_ was observed in the 0.2% FL individuals (**Figures 4A, B**, **10B**).

The OJIP kinetic curve reflects the degree of damage to PSII under light stress (Kumar et al., 2020; Lysenko et al., 2021). The appearance of the K-phase in OJIP is related to the injury of PSII donor side, particularly the OEC (Oxygen-evolving complex) (Zhang et al., 2016; Kumar et al., 2020). However, evidence is accumulating that K-phase is observed when plants are exposed to environmental stress, and K-phase are more pronounced in short-term stressed plants compared with long-term stressed individuals (Pagliano et al., 2006; Tóth et al., 2007). The appearance of the K-phase and the high value of *W*_k_ was obtained in *P. notoginseng* grown under long-term 0.2% FL condition (**Figures 10**, **11B**; *P < 0.05*), and this has been confirmed in *Rosa hybrida* grown under long-term drought stress (Pinior et al., 2005). These results indicate that electron transport is inhibited from electron donor of PSII to the reaction center in low-light-grown individuals, which in turn lead to the OEC injury of PSII donor side. *M*_o_, *Ψ*_o_, *V*_J_ and *φE*_o_ mainly reflects changes in PSII acceptor side (Ayyaz et al., 2020; Kumar et al., 2020; Khan et al., 2021). *V*_J_ and *M*_o_ were increased, and*Ψ*_o_ was decreased in 0.2% FL-grown *P. notoginseng* compared with other counterparts (**Figures 10B**, **11A**), implying that PSII reaction center is closed, a large amount of oxidized Q_A_ is accumulated and the electron transport after Q_A_ is inhibited, consequently resulting in a damage to the acceptor side of the PSII. Nevertheless, the increase in *V*_J_ and *W*_k_ reflects the degree of damage to the acceptor side and the donor side of PSII, respectively (Lu and Zhang, 2000). A similar effect has been observed in *Glycine max* and *Zea mays* grown under environmental stress (Li et al., 2009a; Li et al., 2009b). *V*_J_ and *W*_k_ were significantly increased in 0.2% FL compared with other counterparts, but the increase of *V*_J_ was larger than that of *W*_k_ (**Figures 11A, B**). Anyways, PSII acceptor side is more readily damaged than the donor side in *P. notoginseng* grown under low light condition.

### Cyclic electron flow around PSI protects PSI from damage under long-term light stress

Y(I)/Y(II) was activated earlier when PPFD was higher than 36 μmol·m^-2^·s^-1^ in when *P. notoginseng* were exposed to high light and low light condition (29.8% FL, 0.2% FL; **Figure 7A**), but ETRI - ETRII in 29.8% FL plants was consistently higher than in 0.2% FL plants (**Figure 4C**). These results imply that ΔpH and ATP might be enhanced in high-light-grown *P. notoginseng* compared with the counterparts as has been suggested by Miller et al. (2020). In addition, high ΔpH not only decelerates the damage to PSII by protecting the OEC, but also protect PSI by regulating electron transport from PSII to PSI (Takahashi et al., 2009; Tikkanen et al., 2015). Similarly, cyclic electron flow around PSI plays an essential role in photoprotection for *P. henryi*, *C. cerasoides* and *Phaeodactylum tricornutum* under high-light (Huang et al., 2017; Yang et al., 2019b; Zhou et al., 2020; Sun et al., 2021). ETRI - ETRII, NPQ, ETRI and ETRII were increased, *P*_m_ was substantially reduced in the 29.8% FL plants (**Figures 3A**, **4C**, **6A**), and Y(NPQ), NPQ and Y(ND) have a positive correlation with ETRI - ETRII (**Figure 8**), suggesting that cyclic electron flow around PSI protects PSI and PII from damage by enhancing thermal dissipation capacity and regulating P700^+^ redox state and electron transport in high-light-grown individuals.

Cyclic electron flow around PSI also shows photoprotection in plants exposed to low light (Laisk et al., 2005; Huang et al., 2011; Huang et al., 2012a; Huang et al., 2012b; Huang et al., 2019; Flannery et al., 2021). The maximum values of Y(NPQ), NPQ and Y(ND) were recorded in 0.2% FL-grown plants when ETRI - ETRII is lower (**Figure 8**). High Y(NPQ), NPQ and Y(ND) depend on cyclic electron flow around PSI to produce ΔpH in low-light-grown plants (Munekage et al., 2004). ETRI - ETRII was reduced in the 0.2% FL plants when PPFD is above the value of 272 μmol·m^-2^·s^-1^ (**Figure 4C**), indicating that cyclic electron flow around PSI could not build up a sufficient ΔpH to protect PSII from photodamage in low-light-grown *P. notoginseng*. Severe photoinhibition to PSII would limit the transport of electrons from PSII to PSI, which in turn prevents damage to PSI (Huang et al., 2015). PSII activity and ETRII were drastically decreased when plants were exposed to low light (1.4% FL & 0.2% FL; **Figures 3B**, **4B**), but *P*_m_ was relatively stable (**Figure 3A**). The results obtained herein suggest that severe photoinhibition to PSII protects PSI from photodamage in low-light grown *P. notoginseng*. Overall, cyclic electron flow around PSI cannot completely protect PSII from damage under low light stress, but can prevent PSI photodamage.

## Conclusions

**Figure 13:**
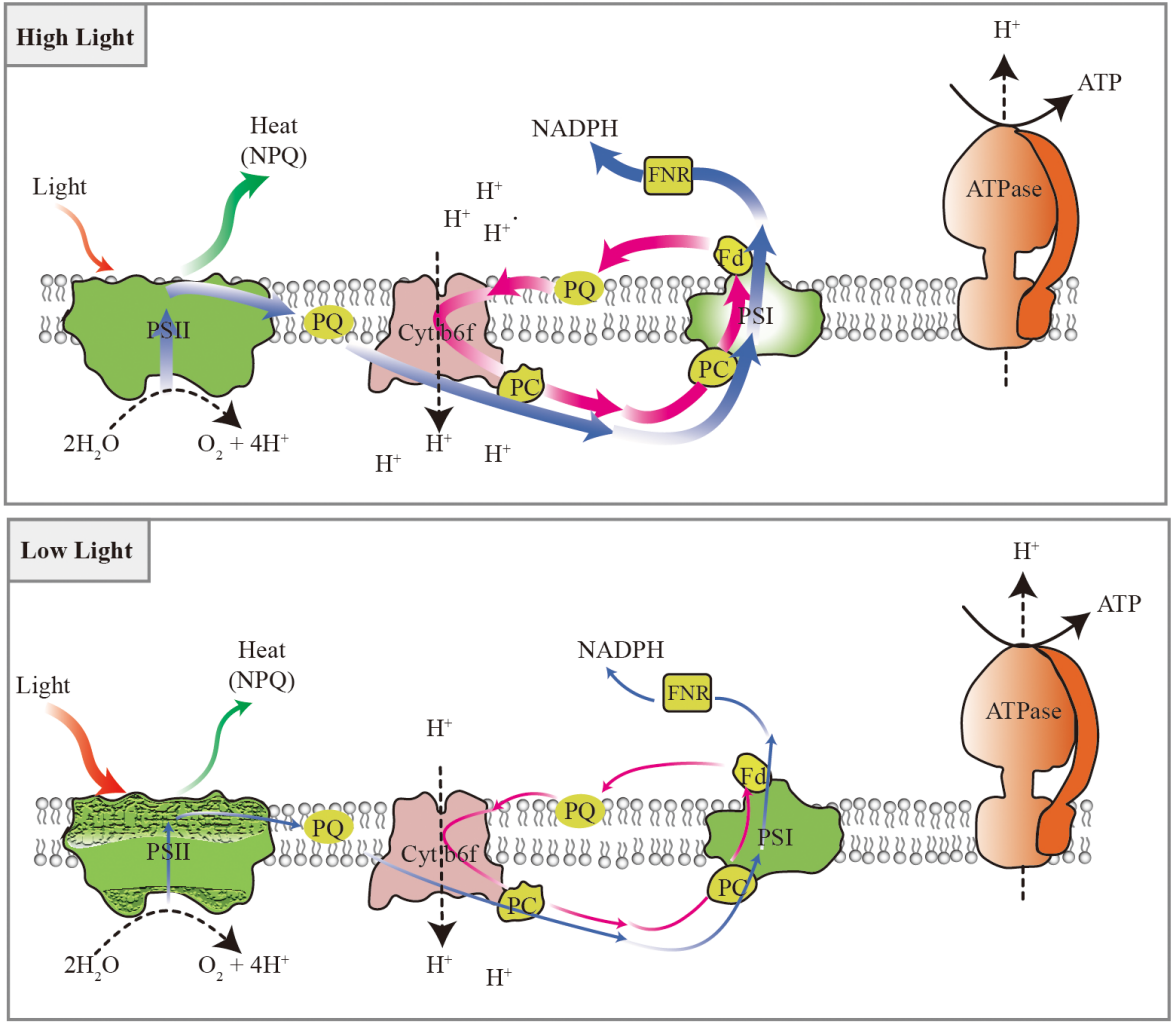
Photosynthetic adaptive strategies of the shade-tolerant species *P. notoginseng* grown under long-term light stress. Energy dissipation through NPQ predominates in response to high light, electron transport plays an important role in utilizing excess light energy, and the moderate photoinhibition of PSII and higher cyclic electron flow around PSI might avoid the damage of the PSI under high light. The absorbed light energy cannot be effectively dissipated and utilized through NPQ and electron transport under low light. Cyclic electron flow around PSI also cannot completely protect PSII from damage under low light. Blue arrows represent linear electron transport, magenta arrow represents cycle electron transport, red arrows represent absorbed light energy, green arrows represent the capability to dissipate heat, craquelure represent the damage of photosystem. The thickness of the lines represents the strength of electron transport, light energy absorption, and heat dissipation. The black dotted line indicates the transport pathway of H^+^. The black solid line indicates the synthetic path of ATP.

A model of photosynthetic adaptive strategies was proposed in the typically shade-tolerant species, such as *P. notoginseng*, grown under long-term light stress (**Figure 13**). The energy dissipation through NPQ predominates in high-light-grown shade-tolerant species. Meanwhile, moderate photoinhibition to PSII and high cyclic electron flow around PSI might avoid the damage to PSI in high-light-grown shade-tolerant species. However, absorbed light energy cannot be effectively dissipated and utilized through NPQ and electron transport in low-light-grown shade-tolerant species. Additionally, cyclic electron flow around PSI also cannot completely protect PSII from damage in low-light-grown shade-tolerant species. PSI photoinhibition is the underlying sensitivity of the shade-tolerant species to high light, and the photodamage to PSII acceptor side might cause the shade-tolerant species to be unsuitable for long-term low light.

## Data availability statement

The original contributions presented in the study are included in the article/**Supplementary Material**. Further inquiries can be directed to the corresponding authors.

## Author contributions

All authors contributed to the conception and design of the study. ZC and J-WC planned and designed the experiments. ZC and X-ZX measured photosynthetic data. ZC, J-YZ, and H-MW analyzed the photosynthetic data. ZC and S-PS plotted the graph. J-WC supervised the data acquisition. ZC, T-XA, and J-WC drafted the manuscript. All authors contributed to the article and approved the submitted version.

## Glossary

**Table:**

*ABS/RC*	Absorption flux per RC
CAT	Catalase
CEF	Cycle electron flow
*C_i_*	Intercellular CO_2_ concentration
Cond	Stomatal conductance
*DI_o_/RC*	Energy dissipation per RC
ETRI	Electron transport rate of PSI
ETRII	Electron transport rate of PSII
*ET_o_/RC*	Trapping energy used for electron transport per RC
*F*_o_	The minimum fluorescence after darkadaptation;
*Fm*	The maximum fluorescence after dark-adaptation
*F*_o_`	The minimum fluorescence after light-adaptation
*F*_m_`	The maximum fluorescence after light-adaptation
*F*_s_	Dark-adapted steady-state fluorescence
*F*_t_	Relative fluorescence intensity at different points of time;
*F*_v_/*F*_m_	The maximum quantum yield of photosystem II
LCP	Light compensation points
LSP	Light saturating points
*M*_o_	Approximated initial slope of fluorescent transient
NPQ	Non-photochemical quenching in PSII
1-*qP*	Redox poise of the primary electron acceptor of PSII
*PI_ABS_*	Performance index for energy conservation from photons absorbed by PSII antenna to the reduction of Q_B_
*P*_m_	The maximum photo-oxidation ${\text{P}}_{700}^{+}$
*P*_max_	The maximum CO_2_ assimilation rate
*P*_n_	Net photosynthesis rate
POD	Peroxidase
PPFD	Photosynthetic photon flux density
PSI	Photosystem I
PSII	Photosystem II
*R*_d_	Dark respiration rates
SOD	Superoxide dismutase
Tr	transpiration rate
*TR_o_/RC*	Trapping flux leading to Q_A_ reduction per RC;
*V_J_*	Relative variable fluorescence at J-step (2 ms)
*W_K_*	Ratio of the variable fluorescent F_K_ occupying the F_J_-F_O_ amplitude
Y(I)	Effective quantum yield of PSI
Y(ND)	Donor side limitation of PSI
Y(NA)	Acceptor side limitation of PSI
Y(II)	Effective quantum yield of PSII photochemistry
Y(NPQ)	Yield of regulated energy dissipation of PSII
Y(NO)	Yield of non-regulated energy dissipation of PSII
*φD*_o_	Quantum yield for thermal dissipation
*φE_o_*	Quantum yield for electron transport (t = 0)
*φP_o_*	The maximum quantum yield for primary photochemistry (t = 0)
*ψ_o_*	Probability that a trapped exciton moves an electron into the electron transport chain beyond ${\text{Q}}_{A}^{-}$ (t = 0).
