# Supplementary material for: Responses of photosystem to long-term light stress in a typically shade-tolerant species Panax notoginseng
Source: Front Plant Sci. 2023 Jan 12;13:1095726. doi: 10.3389/fpls.2022.1095726 (PMC9878349; doi:10.3389/fpls.2022.1095726)

**LEGEND FOR FIGURES**

**Figure S1.** Diurnal variation of photosynthetic photon flux density (PPFD). **(A)** Diurnal variation of PPFD under full sunlight (FL). **(B)** Diurnal variation of PPFD under 29.8% FL. **(C)** Diurnal variation of PPFD under 11.5% FL. **(D)** Diurnal variation of PPFD under 9.6% FL. **(E)** Diurnal variation of PPFD under 5.0% FL. **(F)** Diurnal variation of PPFD under3.6% FL. **(G)** Diurnal variation of PPFD under 1.4% FL. **(H)** Diurnal variation of PPFD under 0.2% FL. Continuous measurement for 3 days, values for each point were means (*n* = 3).

**Figure S2.** Leaf anatomical characteristics of *Panax notoginseng* under light regimes.

**Figure S3.** The growth environment of *Panax notoginseng* in production.

**Figure S1**

**
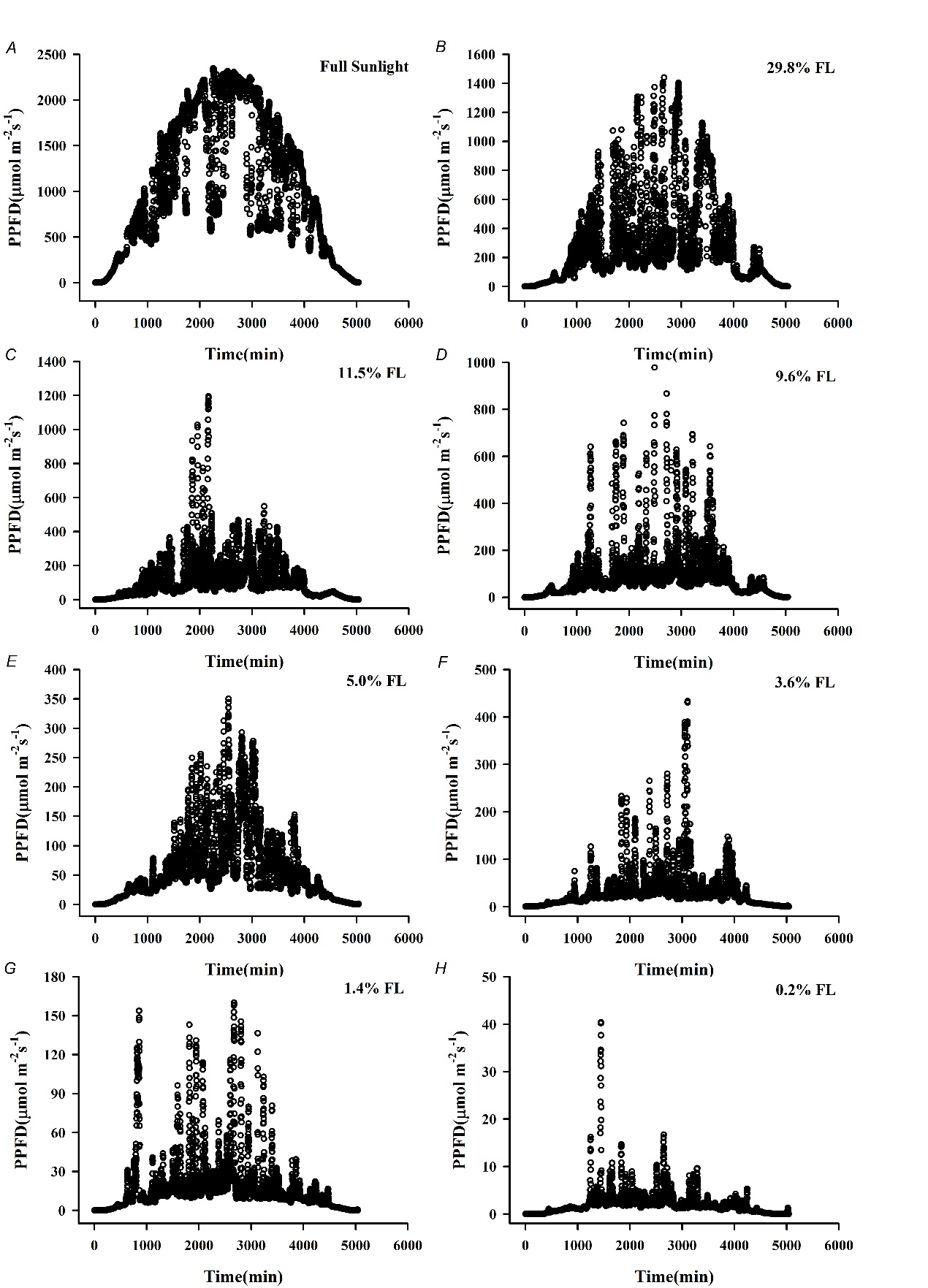
**

**Figure S2**


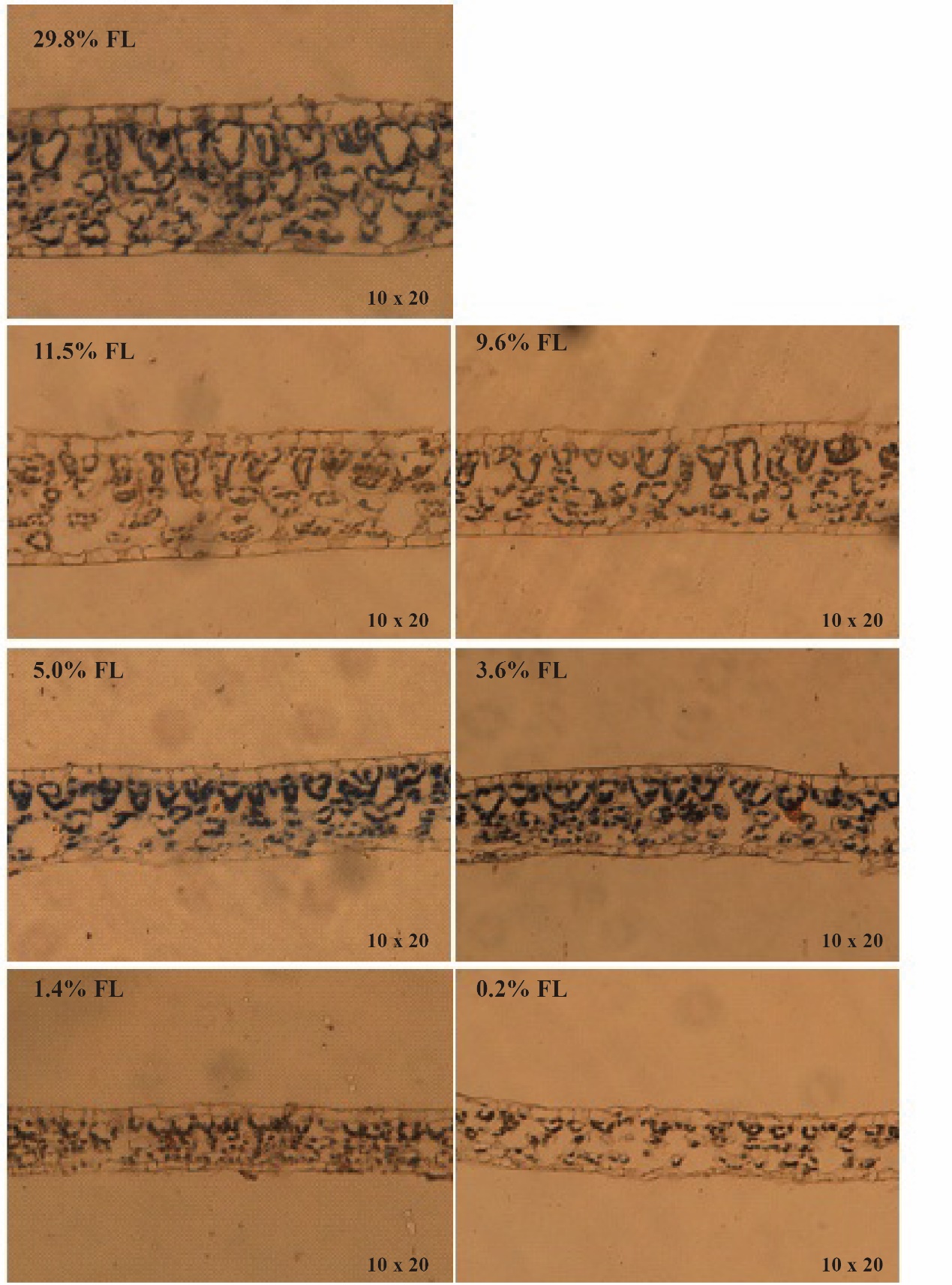


**Figure S3**


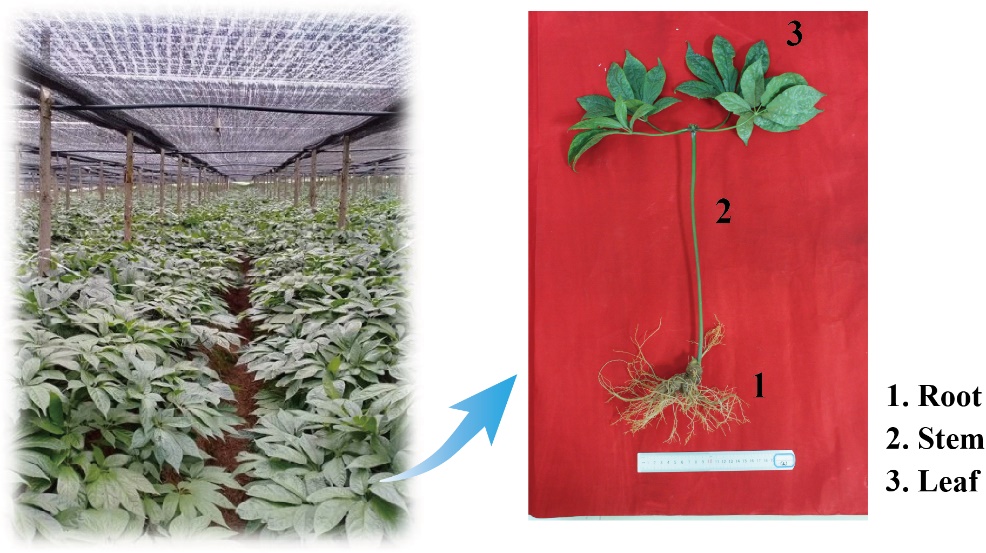

Supplement: Supplementary file 1 [file DataSheet_1.docx]
