# Supplementary material for: Responses of photosystem to long-term light stress in a typically shade-tolerant species Panax notoginseng
Source: Front Plant Sci. 2023 Jan 12;13:1095726. doi: 10.3389/fpls.2022.1095726 (PMC9878349; doi:10.3389/fpls.2022.1095726)
Supplement: Supplementary file 2 [file Table_1.docx]

**Table S1** Formulae and term used in the analysis of the chlorophyll (Chl) *a* fluorescence transient (OJIP) by JIP-test (Yusuf et al., 2010)

| Formulae and terms | Deﬁnitions |
| --- | --- |
| *F*_O_ = *F*_20 µs_ | Fluorescence at time t after onset of actinic illumination |
| *F*_K_ = *F*_300 µs_ | Fluorescence value at 300 µs |
| *F*_J_ = *F*_2 ms_ | Fluorescence value at the J-step (2 ms) of OJIP |
| *F*_I_ = *F*_30 ms_ | Fluorescence value at the I-step (30 ms) of OJIP |
| *F*_P_ = *F*_M_ | Fluorescence value at the peak of OJIP test |
| *V*_J_ = ( *F*_J_ - *F*_O_) / ( *F*_M_ - *F*_O_) | Relative variable fluorescence intensity at the J-step |
| *M*_o_ = 4 × (*F*_300μs_ - *F*_O_) / *F*_v_ | Approximate value of the initial slope of ﬂuorescence transient curves |
| *ϕP_o_* = *TR_o_* */ABS* = (*F*_M_ - *F*_O_) / *F*_M_ | Maximum quantum yield for primary photochemistry, namely FV/FM |
| *ABS/RC* = *M*_o_ × (1 / *V*_J_) × (1 / *ϕP_o_*) | Absorption flux per RC |
| *TR_o_/RC* = *M*_o_ × (1 / *V*_J_) | Trapped excitation ﬂux (leading to QA reduction) per RC |
| *ψ_o_* = *ET_o_ /TR_o_* = 1 - *V*_J_ | Probability that a trapped exciton moves an electron into the electron transport chain beyond Q_A_- (at *t*=0) |
| *ET_o_/RC* = *M*_o_ × (1/ *V*_J_) × *ψ*_o_ | Electron transport ﬂux (further than Q_A_-) per RC |
| *DI_o_/RC* = *ABS/RC*-*TR_o_/RC* | Dissipated energy flux per CS (at *t*=0) |
| *ϕE_o_* = *ET_o_/ABS* = (*F*_V_ / *F*_M_) × (1 - *V*_J_) | Quantum yield of the electron transport ﬂux from Q_A_ to Q_B_ |
| *ϕD_o_* = 1 - *ϕP_o_* | Quantum yield for thermal dissipation |
| *PI_ABS_* = (*RC/ABS*) × [*ϕP_o_* / (1-*ϕP_o_*)] × [*ψ_o_* / (1-*ψ_o_*)] | PI (potential) for energy conservation from exciton to the reduction of intersystem electron |
| *W*_K_ = (*F*_K_ - *F*_O_) / (*F*_J_ - *F*_O_) | Ratio of the variable fluorescent *F*_K_ occupying the *F*_J_-*F*o amplitude. |

Note: the calculation of each parameter is based on the method described by Yusuf et al. (2010). Subscript “o” indicates that the parameter refers to the onset of illumination
